# How to treat fungal infections in ICU patients

**DOI:** 10.1186/s12879-015-0934-8

**Published:** 2015-05-02

**Authors:** Dimitrios K Matthaiou, Theodora Christodoulopoulou, George Dimopoulos

**Affiliations:** Department of Critical Care, University Hospital ATTIKON, Medical School, University of Athens, 1 Rimini str, Haidari-Athens, 12462 Greece

**Keywords:** Candida, Aspergillus, ICU, Critical Care, Fungal infections

## Abstract

Fungal infections represent a major burden in the critical care setting with increasing morbidity and mortality. Candidiasis is the leading cause of such infections, with *C. albicans* being the most common causative agent, followed by Aspergillosis and Mucormycosis. The diagnosis of such infections is cumbersome requiring increased clinical vigilance and extensive laboratory testing, including radiology, cultures, biopsies and other indirect methods. However, it is not uncommon for definitive evidence to be unavailable. Risk and host factors indicating the probability of infections may greatly help in the diagnostic approach. Timely and adequate intervention is important for their successful treatment. The available therapeutic armamentarium, although not very extensive, is effective with low resistance rates for the newer antifungal agents. However, timely and prudent use is necessary to maximize favorable outcomes.

## Introduction

Fungal infections represent a major burden in the critical care setting with increasing morbidity and mortality. Candida infections, Aspergillus infections and Mucormycosis caused by members of the Mucorales order are the most common ones. Each one of them requires a different, although similar, diagnostic and therapeutic approach. The purpose of this review is to provide a practical guide on the basic aspects of each of these infections in the critical care setting.

## Review

### Candidiasis

Candidiasis is the leading cause of fungal infections in Intensive Care Unit (ICU) patients, with *C. albicans* being the most common causative agent [1,2]. Even though *Candida* species is a part of the normal human flora, a small percentage can cause disease: 1) Candidaemia with or without endophthalmitis, 2) Disseminated haematogenous infections with deep organ involvement 3) Chronic disseminated candidiasis, most commonly found in haematological patients [3,4]. *Candida* bloodstream infections (BSIs) are a great proportion of nosocomial fungal infections and represent an important cause of morbidity and mortality in ICU patients [5,6]. Additionally, the true incidence of invasive candidiasis may be higher than estimated because of the small percentage of positive cultures obtained and the difficulty in making a diagnosis of invasive candidiasis without candidaemia [7,8]. There are several risk factors for the development of invasive disease (Table 1), such as the colonization of the gastrointestinal tract, disruption of the mucosa, neutropenia or immunosuppression, the increased use of medical procedures, and poor hygiene of the health care personnel [9].

**Table 1:** **Risk factors associated with** ***Candida spp*** **infections in ICU**

***Candida*** **species**	**Risk factors**
*C. albicans*	Prolonged ICU stay, Corticosteroids, prolonged antimicrobial use, chemotherapy, immunosuppressive agents, Diabetes mellitus, Advanced age, Central venous catheter, Gastrointestinal surgery, Total parenteral nutrition, Pancreatitis, Neutropenia, High disease severity score (APACHE II > 20), Renal replacement therapy, Malnutrition, Multiple site colonization, Major trauma, burns over 50% of body sites
*C. glabrata*	Elderly, malignancies, total parenteral nutrition, central venous catheter, solid organ transplantation, antibiotics (piperacillin/tazobactam, vancomycin), exposure to fluconazole
*C. parapsilosis*	Second most common isolated strain in children, central venous catheter or implanted devices, total parenteral nutrition
*C. tropicalis*	Hematological patients, neutropenia
*C. krusei*	Hematological malignancies, neutropenia, recent gastrointestinal surgery, use of piperacillin/tazobactam, vancomycin, prior exposure to fluconazole
*C. guiillermondi*	Intravascular catheters

The most common pathogenic *Candida spp* is *C. albicans* followed by *C. parapsilosis*, *C. glabrata* and *C. tropicalis*. Less common pathogenic species are *C. krusei*, *C. dublinensis*, *C. guiillermondi*, *C. kefir*, *C. lusitaniae* and *C. rugosa*. A shift was recently observed to non-albicans species and an expanding list of former non-pathogenic species because of the increase in the number of vulnerable population and the ability of isolation of new species in the laboratory [10-12].

Undoubtedly, the early diagnosis and treatment of invasive candidiasis is important but is often not an easy task because of the comorbidities and the delay in obtaining positive cultures. Candida colonization is referred to as a risk factor for developing invasive candidiasis [13,14]. Several studies have presented the use of several clinical scores by using risk factors alone or in combination with sites of colonization in an attempt to identify patients at risk who might benefit from antifungal prophylaxis treatment. The widely used *Candida Colonization Index* (CCI) defined as the ratio of the number of culture positive surveillance sites for *Candida* spp. over the number of sites cultured. If the CCI is greater than 0.4 preemptive antifungal therapy should be commenced [15]. Additionally, a recent study presented the Candida Score (CS), which is based on the following risk factors: surgery upon admission, total parenteral nutrition, severe sepsis and multifocal colonization. A CS above 2.5 identifies high-risk patients who might benefit from antifungal prophylaxis treatment [16].

#### Laboratory diagnosis

There are various laboratory methods aiming at early and accurate diagnosis of invasive candidiasis. Blood cultures have a sensitivity of up to 70%, but they have a long incubation time and they are often negative in deep-sited candidiasis and when fluconazole prophylaxis is used. Serological tests using components of the fungal cell wall such as galactomannan and 1,3-B-d-glucan or antibodies against galactomannan antigen are specific but they lack in sensitivity. Real time polymerase chain reaction, although it has a sensitivity of 60% in diagnosing candidemia, it is invaluable in diagnosing deep-sited candidiasis with a negative blood culture. Lately, newer diagnostic tools were introduced such as matrix-assisted laser desorption ionization time of flight mass spectrometry, molecular detection of fungi in blood and fluorescence in situ hybridization promising an accurate and faster diagnosis [17-19]. Regarding invasive candidiasis, antigen and antibody assays against mannan are used and recommended by the European Society of Clinical Microbiology and Infectious Diseases (ESCMID) guidelines [20]. It should be noted that the MALDI-TOF currently is the most promising method for rapid identification once a fungal organism has been isolated.

#### Empirical and preemptive treatment

It is difficult to recommend the early antifungal therapy to all patients even though it may lead to improved clinical outcomes due to increased costs, toxicity and ecological pressure for antifungal resistance. Antifungal prophylaxis may be useful where there are risk factors or increased local incidence rates. Patients that may benefit from antifungal prophylactic treatment with fluconazole against invasive candidiasis are those who recently underwent abdominal surgery and had recurrent gastrointestinal perforations or anastomotic leakage [20]. In patients with multifocal candida colonization who have clinical risk factors for infection but are in stable condition, preemptive therapy is currently not indicated. In patients with refractory fever receiving broad-spectrum antibacterial therapy, empirical antifungal therapy may be administered and a thorough search for alternate causes should be performed [21].

#### Documented candidiasis

Antifungal drugs for the treatment of candidiasis include polyenes (amphotericin B deoxycholate, liposomal amphotericin B), triazoles (fluconazole, itraconazole, voriconazole, posaconazole), echinocandins (caspofungin, anidulafungin, micafungin) and flucytosine (administered mostly by oral formulation; however it is also available as intravenous formulation in some countries).

Amphotericin B deoxycholate possesses fungicidal properties by interacting with the membrane sterol, increasing the permeability of the cell membrane and allowing leakage of the fungus cell components resulting to its death. It demonstrates high minimum inhibitory concentrations for *C. glabrata* and *C. kruzei* and it has got a narrow therapeutic window. Its lipid formulations retain the activity of the parent drug, exhibit better safety profile, but they more costly. The main adverse events include nephrotoxicity, hepatotoxicity, anemia, and thrombocytopenia. Nephrotoxicity and acute infusion related reactions are the most prominent and the most frequent adverse events. The recommended dosages are 0.5-1 mg/kg daily for amphotericin B and 3–5 mg/kg daily for its liposomal formulations.

All triazoles are fungistatic and they inhibit cytochrome P450 enzymes. Fluconazole has the greatest penetration into the CSF and the vitreous body. Voriconazole is not recommended in patients with clearance lower than 50 ml/min or under hemodialysis due to possible accumulation of cyclodextrin and posaconazole is not available in intravenous formulation and can be used in neutropenic patients with leukemia. They interact with several drugs increasing their concentrations (such as cyclosporine, warfarin, tacrolimus, carbamazepine) and they also increase also the serum levels of fentanyl and midazolam through competitive inhibition. The recommended intravenous dosages are a loading dose of 12 mg/kg followed by a daily dose of 6 mg/kg for fungustatin and 2 loading doses of 6 mg/kg every 12 hours followed by 3-4 mg/kg every 12 hours for voriconazole. Dose adjustments in renal failure should be done. Itraconazole and posaconazole, although active against Candida, they have no label against invasive candidiasis.

Echinocandins (caspofungin, micafungin, anidulafungin) inhibit the synthesis of 1,3-B glucan and they are fungicidal against *Candida* spp. The active drug is not excreted in urine, thus they are not used in patients with candiduria, and it presents high MIC for *C. parapsilosis.* They possess a favorable therapeutic profile with few drug interactions and minor toxicity. The recommended dosing regimens are a loading dose of 70 mg followed by 50 mg daily for caspofungin, a loading dose of 200 mg followed by 100 mg daily for anidulafungin and 100 mg daily for micafungin [22,23]. It should be noted that caspofungin should not be used for the prophylaxis against invasive candidiasis, as it was not found to be superior to placebo in a recent multicenter double blind randomized controlled trial [24].

Candida infections in the ICU may be i) suspected or ii) documented usually by obtaining positive cultures.

i) Suspected candidiasis: if the patient has risk factors for candida infection, prophylaxis treatment may be initiated usually with fluconazole. Risk factors in combination with positive serological markers may indicate patients eligible for pre-emptive treatment.
ii) Documented candidiasis: therapy should be commenced at the moment the blood cultures show the growth of yeast without waiting for the species identification and the results of susceptibility testing. Recent ESCMID guidelines suggest the use of echinocandins first, especially in unstable patients. (Table 2) [20]. Current IDSA guidelines suggest the use of fluconazole, echinocandins and amphotericin B or its lipid formulations.

**Table 2:** **Treatment of documented invasive candidiasis**

	**First line**	**Alternatives**
ESCMID (2012)	- Anidulafungin 200 mg loading dose, then 100 mg/day	- Liposomal amphotericin B 3 mg/kg
	- Caspofungin 70 mg loading dose, then 50 mg/day	- Voriconazole 3–6 mg/kg/day
	- Micafungin 100 mg	- Fluconazole 400–800 mg
		- Amphotericin B lipid complex 5 mg/kg
IDSA (2009)	*Stable patients*	- Lipid formulations of amphotericin B 3–5 mg/kg daily - Amphotericin B deoxycholate 0.5-1 mg/kg daily
	- Fluconazole 800 mg loading dose, then 400 mg	
	*Unstable patients or recent use of fluconazole*	
	- Anidulafungin 200 mg loading dose, then 100 mg/day	- Voriconazole 400 mg twice daily for 2 doses, then 200 mg twice daily
	- Caspofungin 70 mg loading dose, then 50 mg/day	
	- Micafungin 100 mg/day	

In the presence of candida infection, consecutive blood cultures should be obtained and the length of treatment is defined to 14 days after the last positive blood culture. It is recommended that central venous catheters or implanted devices (pacemakers and implantable defibrillators) should be removed and a fundoscopic examination should be performed in order to exclude endocular infection. In the presence of localized candidiasis the suggested treatment, according to IDSA guidelines, is presented in Table 3 [25].

**Table 3:** **Treatment of localized candidiasis**

	**Primary therapy**	**Alternative therapy**
Pyelonephritis	Fluconazole 200–400 mg for 14 days	Amphotericin B deoxycholate 0.5-0.7 mg/kg daily +/− flucytosine 25 mg/kg four times daily or flucytosine alone for 14 days
Osteomyelitis	Fluconazole 400 mg daily for 6–12 months	Echinocandin* or Amphotericin B deoxycholate 0.5-1 mg/kg daily for several weeks and then fluconazole for 6–12 months
	Lipid formulations of amphotericin B 3–5 mg/kg daily for several weeks, then fluconazole for 6–12 months	
Septic arthritis	Fluconazole 400 mg daily for at least 6 weeks	Echinocandin* or Amphotericin B deoxycholate 0.5-1 mg/kg daily for several weeks and then fluconazole
	Lipid formulations of amphotericin B 3–5 mg/kg daily for several weeks, then followed by fluconazole	
CNS candidiasis	Lipid formulations of amphotericin B 3–5 mg/kg +/− flucytosine 25 mg/kg four times daily for several weeks, followed by fluconazole 400–800 mg daily until the resolution of symptoms	Fluconazole 400–800 mg daily for patients intolerant to lipid formulations of amphotericin B
Endophthalmitis	Amphotericin B deoxycholate 0.7-1 mg/kg with flucytosine 25 mg/kg four times daily for at least 4–6 weeks along with surgical intervention for severe cases	Lipid formulations of amphotericin B 3–5 mg/kg daily
		Voriconazole 6 mg/kg twice daily for two doses, then 3–4 mg/kg twice daily
		Echinocandin*
Endocarditis	Lipid formulations of amphotericin B 3–5 mg/kg +/− flucytosine 25 mg/kg four times daily	Valve replacement is strongly recommended, otherwise chronic suppression with fluconazole 400–800 mg daily is recommended. Lifelong suppressive therapy for prosthetic valve endocarditis if valve cannot be replaced is recommended.
	Amphotericin B deoxycholate 0.6-1 mg/kg +/− flucytosine 25 mg/kg four times daily	
	Echinocandin**	

*Anidulafungin 200 mg loading dose, then 100 mg/day; Caspofungin 70 mg loading dose, then 50 mg/day; Micafungin 100 mg/day.

**For endocarditis higher doses of echinocandins may be required such as Anidulafungin 100–200 mg/day; Caspofungin 50–150 mg/day; Micafungin 100–150 mg/day.

### Aspergillosis

*Aspergillus* is a fungal genus encompassing hundreds of molds, the most clinically important of which are *A. fumigatus*, *A. flavus, A. terreus, A. niger, and A. nidulans.* Recently, *Neosartorya udagawae* has also emerged with increased clinical impact [26,27]. It has a wide environmental distribution, it may be found in the soil, water, air and decomposing organic matter, and may colonize or cause infection mostly in immunocompromised patients.

#### Epidemiology, incidence and outcomes

In the critical care setting, Aspergillus may harbor in the ICU ventilation and water systems that have been poorly maintained, as well as in various equipment. However, there is a difficulty in discriminating between colonization and infection when they are isolated from the patient. Incidence varies depending on the immune status of the patients. Virtually any organ may be affected by *Aspergillus* species, with sinopulmonary involvement as invasive pulmonary aspergillosis being the most common [28]. The lowest incidence for invasive aspergillosis (IA) is reported in patients with HIV and in patients with hematologic malignancy, which is 0.4% [29]. The incidence in patients with solid organ transplants vary from 0.1 to 2.4%, while in patients with stem cell transplants, vary from 0.5 to 3.8% [30]. In patients with hematopoietic stem cell transplantation incidence rates are about 5% for autologous and 5-10% for allogeneic transplantation, respectively [31,32].

Mortality rates also vary. The overall mortality rates of Aspergillosis are about 17%, based on US national data, but mortality is higher in cases of Aspergillus pneumonia and in immunocompromised patients with IA [33]. Specifically, in Aspergillus pneumonia mortality rises to 25%, while in patients with blood and lymphoid tissue malignancies, bone marrow transplant recipients, and liver transplant recipients, mortality rates of IA are 49%, 80%, and 90%, respectively [33,34].

It should be noted that the immune status of critically ill patients, as well as other underlying conditions, are important determinants of the type of fungal infection they may develop and it is usually of the invasive form. Thus, patients receiving steroids are in increased risk of having cavitating lesions and aspergillomas, and neutropenic patients develop angioinvasive aspergillosis, as neutrophils play a major role in the clearance of Aspergillus [35,36]. Patients who received lung transplantation are often colonized with Aspergillus due to their primary lung disease [37] and are more likely to present with aspergillosis around the anastomoses [38]. Also, involvement of other foci including endocarditis and osteomyelitis has been reported [39,40].

#### Risk factors

Neutropenia was the first risk factor to be recognized for invasive aspergillosis more than forty years ago [41]. The absolute neutrophil number per se is not the sole determinant, as the overall neutrophil functional status plays a major role in the pathogenesis of Aspergillus. However, one should keep in mind that neutropenia and compromised immune functional status usually concur in several conditions.

Following this rough categorization, common neutropenic conditions that may lead to the development of invasive aspergillosis are hematologic malignancies and myeloablative chemotherapy in stem cell recipients [42]. Prolonged duration and degree of neutropenia are important factors of developing the disease. Solid organ transplantation recipients, especially of lung, are at increased risk for invasive aspergillosis, as they receive immunosuppressive therapy and are subjected to environmental exposure to Aspergillus.

Regarding the deterioration of the immune system’s functionality, steroid therapy is a major risk factor as it may be administered either as anti-inflammatory drug in the course of infections or as immunosuppressive treatment during transplantations [43-46]. Other similar conditions associated with invasive aspergillosis are prior antibiotic treatment, AIDS, H1N1 infection, chronic obstructive pulmonary disease, chronic granulomatous disease, and acute renal failure [43,44,47-50]. It is interesting that patients receiving immuno-modulatory drugs, namely TNF-a inhibitors, are also at increased risk for invasive aspergillosis [51].

#### Diagnosis

The diagnosis is based on a triplet consisting of (i) risk and host factors (the latter not being identical to risk factors), (ii) clinical and radiological signs and symptoms that are indicative of the disease and (iii) laboratory testing that proves the existence of Aspergillus either directly or indirectly.

According to the definitions published by the European Organization for Research and Treatment of Cancer/Mycosis Study Group (EORTC/MSG), the utilization of these criteria classify the diagnosis of invasive aspergillosis in proven, probable and possible [52]. For a proven infection, it is required to have a positive culture of an otherwise sterile site or a positive histopathologic or cytopathologic examination. It should be noted that, although very useful in the diagnostic process, clinical symptoms and signs and risk and host factors are not mandatory when the presence of Aspergillus has been demonstrated. For a probable infection, all elements of the aforementioned triplet are required to be positive. However, mycological evidence retrieved from various sites not essentially sterile may be used including bronchoalveolar lavage, bronchial specimens retrieved with brush, sinus aspirates, sputum etc. For a possible infection, a diagnosis can be done when there are appropriate risk/host factors and clinical symptoms and signs, but mycological evidence is lacking.

It should be noted that these definitions were based on the study of immunosuppressed patient. As such, they may not be fully applicable in critically ill patients, who are a different population, although immunosuppressed patients may represent a subset of them. It has been supported that the presence of Aspergillus in that patient setting, irrespective of infection or colonization, indicates a poor prognostic marker [53]. The use of appropriate clinical algorithms may offer further aid in diagnosing invasive aspergillosis and discriminating between infection and colonization with relatively high sensitivity and specificity [54].

The radiological tests that may be used are chest x-rays and CT scanning. The most common signs that may be found are consolidations, and infiltrates and nodules [55]. However, the pathognomonic sign is the “halo sign”, which is found in CT scans as a nodule with a dense center surrounded by ground glass opacity. It should be noted that the latter is mostly found in neutropenic patients, while relevant findings of non-neutropenic patients are nonspecific [56].

Laboratory testing used for the detection of Aspergillus includes biopsies, cultures, detection of galactomannan and β-D-glucan and PCR. As noted before, the isolation of Aspergillus from cultures depends on whether the site of isolation is otherwise sterile, since a positive culture from respiratory tract secretions, irrespective of the method used for isolation, may represent colonization rather than infection [57]. Galactomannan is detected in body fluids and depending on its concentration galactomannan index is calculated. When a certain threshold is surpassed, then a diagnosis of invasive aspergillosis is likely. Although the sensitivity of the method in the serum is limited [58], it seems that galactomannan detection in bronchoscopic material, such as bronchoalveolar lavage, has superior accuracy and is not affected by the immune status of the patient [59]. β-D-glucan is, like galactomannan, a cell wall component, which is also found in body fluids and its presence indicates the probability of infection. PCR, although it is a method with high sensitivity and specificity, is cumbersome and lacks in discrimination power between colonization and infection [60,61]. Quantitative real time PCR may pose as a useful alternative in the diagnosis of invasive aspergillosis [62].

#### Treatment

Treatment of aspergillosis may be classified in empirical, preemptive and definitive. Empirical treatment is the one administered to neutropenic patients at risk with a prolonged febrile period, who have already received broad antibiotic coverage. Preemptive therapy is the one administered when there is evidence of fungal existence without a developed infection. However, its benefit has been questioned compared to empirical treatment [63]. Definitive treatment is the one administered when infection has developed and it is based on antibiogram data. When definitive treatment with a single agent is not successful, combination treatment may be used in the salvage setting [64]. Prophylactic treatment, although administered in neutropenic patients, has not found its place in the critical care setting. Adjunctive therapies including Granulocyte-Colony Stimulating Factor and interferon-gamma may be used. Surgical treatment for the resection of Aspergillus lesion may be an alternative option in selected cases [65].

Although amphotericin B, azoles and echinocandins may be used for the treatment of Aspergillosis, voriconazole is considered as the first line treatment, especially for the invasive pulmonary form. It is administered intravenously in a dosage of 6 mg/kg twice daily as a loading dose and then the dose is lowered to 4 mg/kg twice daily. Amphotericin B is administered intravenously as liposomal amphotericin in a dosage of 3 to 5 mg/kg/day or as its lipid complex in a dosage of 5 mg/kg/day, but has lost its predominance due to the nephrotoxicity it may confer and the newer therapeutic alternatives that have been found [66]. A paradox regarding its reduced toxicity when administered in high doses, although without a subsequent improvement in outcomes, should be noted [67]. From the echinocandin class, only caspofungin has been approved for the treatment of Aspergillosis, but is used when treatment with other agents is not successful or not allowed due to adverse events. It is used mostly when other antifungals have failed and it is administered in combination with amphotericin [68]. It is administered intravenously in a dosage of 70 mg/day as a loading dose and 50 mg/day thereafter [66]. When administered in combination with calcineurin pathway inhibitors, it was found to have increased in vitro activity [69]. Itraconazole and posaconazole is considered second-line treatment option. Posaconazole has been approved as salvage treatment of invasive aspergillosis in the European Union only.

The duration of treatment varies depending on the patient’s immune status. In non-immunosuppressed patients it should last for a minimum of 6 to 12 weeks. In immunosuppressed patients, treatment should be administered as long as the patient is immunosuppressed and should not be stopped until the clinical and radiological resolution of symptoms and signs. Laboratory testing to certify the eradication of the fungus is not suggested. Relapses due to incomplete eradication and lack of sterilization of underlying foci may occur.

### Mucormycosis

Mucorales are saprophytes causing a disease known as mucormycosis. The most common genera include *Rhizopus*, *Mucor* and *Rhizomucor.* Risk factors in the critical care setting include neutropenia, diabetes, malignancy, desferoxamine therapy, renal failure, hematopoietic stem cell transplantation and penetrating trauma [70]. Mortality is high ranging from 35% when no underlying condition is present to 66% in patients with malignancy.

Methods for the diagnosis of mucormycosis are limited. Practically, the sole approach that may provide evidence of Mucorales presence is through histopathology using hematoxylin-eosin, Grocott-methanamine-silver and periodic acid-Schiff stains. PCR use has also been described, but it is not routinely used. Radiological testing rarely provides specific findings [71].

Treatment options are also limited. The mainstay of treatment is amphotericin B in its deoxycholate, lipid complex or liposomal form. The respective dosages are 1 to 1.5 mg/kg/day, 5 to 7.5 mg/kg/day and 5 to 10 mg/kg/day. It should be noted that the deoxycholate form, although the most toxic and with the poorest penetration in the central nervous system, is the only formulation approved for the treatment of mucormycosis. Limited data regarding combination treatment of lipid amphotericin with echinocandins are also available [72]. The combination of deferasirox with lipid amphotericin was found to be highly fungicidal in animal models [73]. However, a recent small phase II trial found that patients with mucormycosis treated with deferasirox had a higher mortality rate at 90 days and did not support a role for initial, adjunctive deferasirox therapy for mucormycosis [74]. Surgical debridement may also be used as adjunctive therapy [75].

The duration of treatment for mucormycosis is not established and should be individualized for each patient. It should be continued until there is resolution of clinical signs and symptoms, radiological findings, if any, and of immunosuppression.

## Conclusions

Fungal infections, although of great importance, are not easy to diagnose and treat in the critical care setting. To limit the mortality toll of such infections, increased clinical vigilance and a multifaceted diagnostic approach is mandatory. The therapeutic armamentarium, although not very extensive, is effective with low resistance rates for the newer antifungal agents. However, timely and prudent use is necessary to maximize favorable outcomes.
